# *Fusobacterium nucleatum* Caused DNA Damage and Promoted Cell Proliferation by the *Ku70*/*p53* Pathway in Oral Cancer Cells

**DOI:** 10.1089/dna.2019.5064

**Published:** 2020-01-08

**Authors:** Fengxue Geng, Yunjia Zhang, Ze Lu, Shuwei Zhang, Yaping Pan

**Affiliations:** Department of Periodontics, School of Stomatology, China Medical University, Shenyang, Liaoning, China.

**Keywords:** OSCC, *Fusobacterium nucleatum*, DNA damage, γH2AX, Ku70

## Abstract

Bacterial infection influences genomic stability and integrity by causing DNA damage, which increases the possibility of tumor initiation and development. We aimed to investigate whether *Fusobacterium nucleatum*, one of the periodontal pathogens, promoted oral squamous cell carcinoma (OSCC) by causing DNA double-strand break (DSB). Tca8113 tongue squamous cell carcinoma cells were infected with *F. nucleatum*. The expression of γH2AX was detected by western blots and immunofluorescence. The proliferation and cell cycle alterations were tested by CCK8 and flow cytometry, respectively. The expression levels of Ku70, p53, and p27 were evaluated by quantitative real-time polymerase chain reaction and western blots. A plasmid was used for the overexpression of Ku70 to verify the possible relationship between Ku70 and p53. We confirmed the presence of DSBs in the response to *F. nucleatum* by detecting the expression of γH2AX. The cell proliferation ability was increased with an accelerated cell cycle while the expression of p27 was decreased. Meanwhile, the expression of Ku70 and wild p53 was downregulated. When Ku70 was overexpressed, the expression of wild p53 in response to *F. nucleatum* infection was upregulated and cell proliferation was accordingly inhibited. We concluded that *F. nucleatum* infection promoted the proliferation ability of Tca8113 by causing DNA damage via the Ku70/p53 pathway.

## Introduction

Oral squamous cell carcinoma (OSCC) is the most common type of head and neck malignancy with a high prevalence and a poor survival rate. It is widely accepted that inflammatory microenvironment is regarded as an important risk factor of all stages of tumor. Approximately 15–20% of malignancies are followed by inflammation that is sustained long before tumor formation (Parkin, 2006; Grivennikov *et al.*, 2010; Greten and Grivennikov, 2019).

Bacterial infections, one of the important inflammatory factors, are being increasingly accepted as a risk factor for various types of cancer including OSCC (Moergel *et al.*, 2013). Based on a recent report, the oral microbiome in OSCC lesions shifts significantly compared to healthy epithelium. Specifically, *Fusobacterium* species are regarded as one of the major components of the oral microbiome in OSCC (Perera *et al.*, 2018). The role of *Fusobacterium nucleatum* in OSCC is being gradually recognized (Binder Gallimidi *et al.*, 2015; Chang *et al.*, 2018; Hsiao *et al.*, 2018).

DNA damage is known to contribute to tumor initiation and development. In addition to traditional environmental factors such as carcinogens and radiations, inflammation is also involved in the regulation of DNA damage response (DDR) genes (Gronke *et al.*, 2019). Specifically, DNA damage and repair are reported to be affected by bacteria, such as *Helicobacter pylori* or *Pneumococci* (Obst *et al.*, 2000; Rai *et al.*, 2016; Shi *et al.*, 2019).

Histone H2A (H2AX) is a meiosis-specific isoform of histone H2A. γH2AX, the phosphorylated form of H2AX, is rapidly and accurately recruited when there is a DNA double-strand break (DSB), the most serious type of DNA damage, and it is applied as a DSB molecular marker (Rogakou *et al.*, 1998; Bach *et al.*, 2017). Once the DNA damage occurs, DSBs are repaired by homologous recombination or nonhomologous end joining (NHEJ). NHEJ, initiated by Ku, can bind to any two DNA break ends by sensing and binding DSBs, which makes it the most common DSB repair pathway in mammalian cells (Mari *et al.*, 2006). Of note, a deficiency in NHEJ results in malignant transformation. Ku is a stable heterodimer structure formed by Ku70 and Ku80 (Puebla-Osorio *et al.*, 2014; Lamaa *et al.*, 2016). Current research has shown that Ku70 participates in DDR signaling through activating cell cycle detection points and initiating apoptosis programs.

Cell cycle disregulation is one of the common features of human cancers (Malumbres and Barbacid, 2009). Cell cycle progression is determined by cyclin-dependent kinases (CDKs) that are activated by binding cyclins and are inhibited by CDK inhibitors (CDKIs). Accumulation of DNA alterations might induce cell cycle progression by abnormal regulations of CDKIs, which results in genomic instability and ultimate oncogenesis (Kastan and Bartek, 2004; Malumbres and Barbacid, 2009). p27, a tumor suppressor, is one of the major members of the CDKI protein family and functions to negatively regulate cell cycle progression (Keles *et al.*, 2003; Breitenstein *et al.*, 2013). Studies have shown that p27 can cause abnormal cell proliferation, and downregulation of p27 is associated with a poor tumor prognosis (Pereira *et al.*, 2013). As recently reported, *F. nucleatum* infection was associated with the activation of cyclin D1, which facilitated intestinal tumorigenesis (Wu *et al.*, 2018; Rubinstein *et al.*, 2019). However, the role of *F. nucleatum* in regulating the expression of CDKIs such as p27 remained investigations.

The Ku complex can bind to DSBs ends with the activation of protein sensors such as p53. To date, the interactions of Ku and p53 remain controversial. As DNA damage occurs, Ku is acetylated in the DNA binding region and its bond with p53 is thereby released, which leads to upregulation of p53 expression and initiation of DNA repair (Lamaa *et al.*, 2016). However, on the contrary, some studies have shown that the NHEJ repair of DSBs is significantly different in colorectal cancer tissues and adjacent normal tissues, revealing a positive correlation between Ku and p53 changes (Komuro *et al.*, 2002; Lu *et al.*, 2015). To date, the role of *F. nucleatum* in DNA damage and the possible association with OSCC has not been clearly elucidated.

In the present study, we established a DNA damage model of OSCC cells infected with *F. nucleatum* at an MOI of 500 and a DSB molecular marker was evaluated. With further investigation, we found that the resulting increased proliferation ability and accelerated cell cycle of OSCC cells in response to DNA damage was dependent on the Ku70/p53 pathway.

## Materials and Methods

### Bacteria and eukaryotic cell culture

Frozen stock of *F. nucleatum* ATCC 25586 (provided by the Department of Oral Biology, Stomatology of China Medical University) was recovered on tryptic soy broth (TSB) agar plates and anaerobically incubated at 37°C for 3–5 days. Appropriate colonies from the plate were resuspended in TSB liquid medium and anaerobically cultured for 16 h before use. *F. nucleatum* in the mid-log phase was adjusted to 1 × 10^9^ CFU/mL (OD = 1) in RPMI 1640 cell culture medium with a spectrophotometer at a wavelength of 600 nm.

Tca8113 tongue squamous cell carcinoma cells were purchased from the Shanghai Institute of the Chinese Academy of Sciences. Cells were routinely cultured in RPMI 1640 medium containing 10% fetal bovine serum, 100 U/mL penicillin, and 100 mg/mL streptomycin and incubated at 37°C, 5% CO_2_.

### Establishment of the DNA damage model

Cells (2 × 10^5^ cells/well) were cultured at 37°C for 24 h. Then, the cells were incubated with fresh medium without penicillin and streptomycin. Actively growing *F. nucleatum* at an MOI of 500 was added to the cell culture plate and cultured for 36 h. The expression of γH2AX was detected at 0, 4, 12, 24, and 36 h, respectively.

### Cell immunofluorescence assay

Actively growing cells were subcultured and inoculated on sterilized glass slides. After the cells were infected by *F. nucleatum* for 24 h, cells on the glass slides were treated with precooled 4% paraformaldehyde for 30 min and 0.2% Triton X-100 at room temperature for 10 min. The cell slides were blocked with 1% BSA for 30 min at room temperature and incubated with a primary antibody against γH2AX (1:1000) overnight at 4°C. The cell slides were then incubated with fluorescent secondary antibody (1:500) for 1 h at room temperature. After being stained with DAPI at room temperature for 5 min, the slides were mounted on glass slides with antifluorescence quenching tablets and observed under a fluorescence microscope. The culture medium and cells in medium without bacterial infection were used as negative controls.

### Cell proliferation assay by CCK-8

Cells were inoculated into 96-well plates (3000/well) and the DNA damage model was constructed 24 h later. At 0, 4, 12, 24, and 36 h, CCK-8 assay solution (10%) was added to each well and incubated at 37°C in the dark for 2 h. The absorbance was then measured at 450 nm using a microplate reader. The culture medium and cells in medium without bacterial infection were used as negative controls.

### Cell cycle analysis by flow cytometry

Cells were starved for 24 h in serum-free medium before infection with *F. nucleatum*. At 0, 4, 12, 24, and 36 h, cells were digested and fixed in 75% ethanol (precooled) overnight at 4°C. Then, the fixed cells were washed with ice-cold PBS and resuspended in mixed media containing propidium iodide (50 μg/mL) and RNase (100 μg/mL) for incubation at 37°C in the dark for 30 min. The cell cycle was determined by a flow cytometer (FACS, Becton-Dickinson, Islandia, NY). The culture medium and cells in medium without bacterial infection were used as negative controls.

### Quantitative real-time polymerase chain reaction

Total RNAs of each group were extracted and quantitative real-time polymerase chain reaction (qPCR) was performed according to the manufacturer's instructions as described previously (Liu *et al.*, 2015). The fold induction of the selected genes was calculated compared using the 2^−ΔΔ^CT method (Schmittgen and Livak, 2008). The primer sequences are shown in the Supplementary (Table S1).

### Western blot assay

Total protein was extracted with RIPA lysates and the protein concentration was determined with a bicinchoninic acid protein assay on ice. An equal amount of sample proteins were separated with 12% SDS-polyacrylamide gel electrophoresis, followed by being transferred to a nitrocellulose filter membrane. After being blocked in BSA medium for 1 h, the blots were probed with the following primary antibodies: anti-γH2AX (1:1000) (Abcam); anti-Ku70 (1:1000) (Santa Cruz Biotechnology); anti-p27 (1:500) (Affinity); anti-wild p53 (1:1000) (Wanleibio Co., Ltd., China); or anti-GAPDH (Proteintech Group) overnight. The blots were washed with TBST three times and exposed to the secondary antibody (1:500) (LI-COR) at room temperature for 1 h. The protein bands were detected with Odyssey CLX (LI-COR) and analyzed with ImageJ software (1.42q).

### Overexpression of Ku70 by plasmid transfection

The overexpression plasmid for Ku70 was constructed by the GenePharma cooperation (Shanghai, China) and the plasmid was extracted according to the instructions of the Rapid Mini Plasmid Kit (Biomed, Beijing, China). Actively growing cells at 60% confluence were used for transfection with Lipo2000 for 24 h. The normally cultured cells, cells transfected with an empty vector and cells treated with Lipo2000 by itself were used as controls. The transfection efficiency was measured by qPCR. After transfection, the proliferation ability induced by *F. nucleatum* and the expression of wild p53 were measured.

### Statistical analysis

The one-way ANOVA-LSD multiple comparison method or a rank sum test was used for statistical analysis. The level of detection was double-sided *α* = 0.05. All of the experiments were repeated at least three times. All statistical results were performed using the statistical analysis software IBM SPSS Statistics version 23. *p* < 0.05 was considered statistically significant.

## Results

### Successful construction of the cellular DNA damage model

**FIG. 1.**
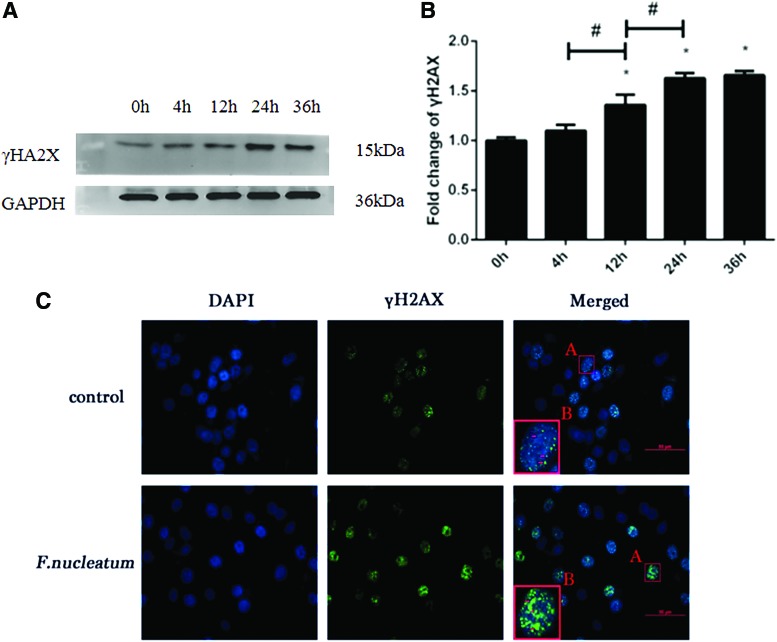
At an MOI of 500, the protein expression of γH2AX was increasingly upregulated from 0 to 36 h **(A)**. The expression of γH2AX was normalized to GAPDH and the relative fold change was compared with the control group **(B)**. The data are presented as the mean ± standard deviation of three independent assays. *Significant difference (*p* < 0.05) compared with the control group (0 h). ^#^Significant difference (*p* < 0.05) compared between the tested groups. After the infection with *Fusobacterium nucleatum* at an MOI of 500 for 24 h, the expression of γH2AX was further analyzed with immunofluorescence **(C)**. The nuclei labeled with DAPI are shown in *blue* and the expression of γH2AX is presented in *green* fluorescence (*red arrow*). As we can see, the number of cells stained with *green* fluorescence and the fluorescence intensity of γH2AX were significantly increased in response to *F. nucleatum* infection. Color images are available online.

In the present cellular model, an MOI of 500 was applied for *F. nucleatum* infection based on the results of a preliminary study (shown in Supplementary Fig. S1). After the cells were infected with *F. nucleatum* at an MOI of 500, western blotting was used to detect the expression of γH2AX (shown in Fig. 1A, B). The expression of γH2AX protein was increased continuously in a time-dependent manner within 36 h, indicating that the DNA damage model of Tca8113 lingual squamous carcinoma cell was successfully constructed. The expression of γH2AX was significantly increased at 24 h, suggesting that DNA damage was significantly aggravated at 24 h (*p* < 0.05). Moreover, based on the immunofluorescence results, the fluorescence intensity of γH2AX at 24 h was increased compared to the blank control group and the number of positive cells was also increased (Fig. 1C). The time point of 24 h was therefore selected for use in the exploration of the molecular mechanisms.

### DNA damage caused by *F. nucleatum* promoted cell proliferation with an accelerated cell cycle

**FIG. 2.**
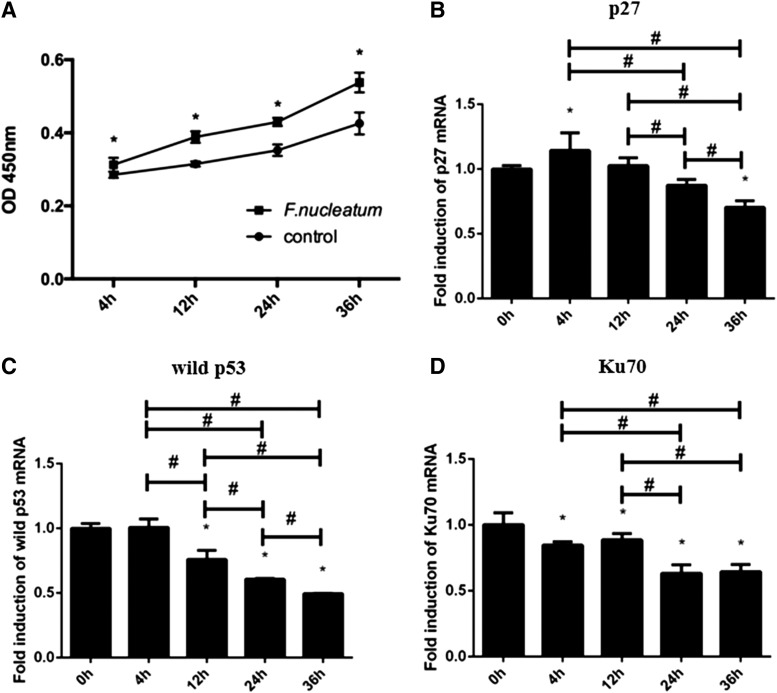
The ability of cell proliferation was increased after *F. nucleatum* infection from 4 to 36 h **(A)**. The effect of *F. nucleatum* infection on the mRNA expression of *p27* **(B)**, wild *p53* **(C)**, and *Ku70* **(D)** was evaluated by qPCR. The data were analyzed according to relative gene expression by 2^−ΔΔCt^. The data are presented as the mean ± standard deviation of three independent assays. *Significant difference (*p* < 0.05) compared with the control group. ^#^Significant difference (*p* < 0.05) compared between the tested groups. qPCR, quantitative real-time polymerase chain reaction.

**FIG. 3.**
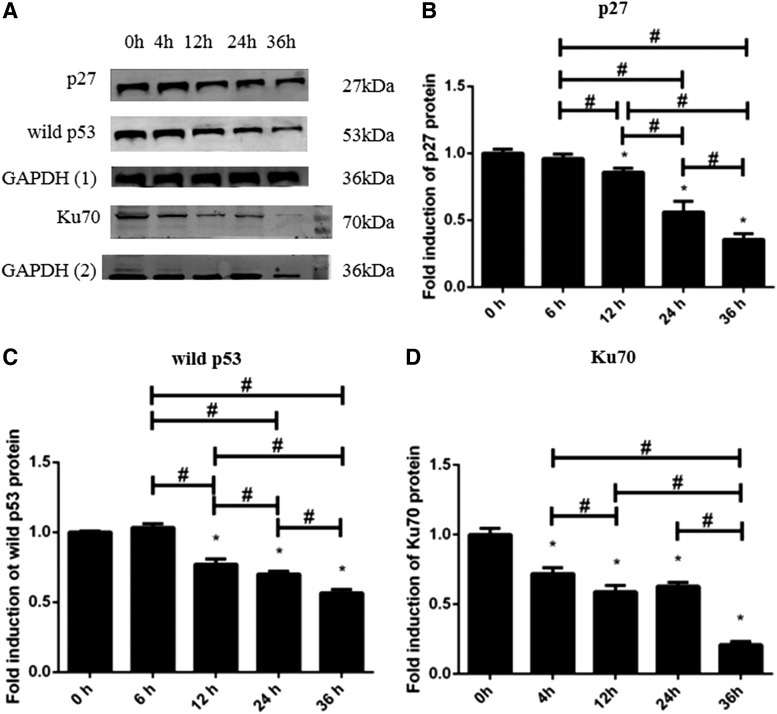
The protein expression of p27, wild p53, and Ku70 in response to *F. nucleatum* infection **(A)**. The expression of p27, wild p53, and Ku70 was normalized to GAPDH and the relative fold change was compared with the control group **(B–D)**. The fold inductions are presented as the mean ± standard deviation of three independent assays. *Significant difference (*p* < 0.05) compared with the control group. ^#^Significant difference (*p* < 0.05) compared between the tested groups.

Cells infected by *F. nucleatum* had an increased proliferative capacity in a time-dependent manner at each time point (*p* < 0.05) (Fig. 2A). The proportion of cells in the G1 phase of the cell cycle was significantly decreased at the time points of 12 to 36 h while the proportion in the S phase was increased from 4 to 24 h. Of note, although no significant alteration was seen in S phase at 36 h, the G2/M phase in the infected cells was significantly higher than that in the control (Table 1). It was found that the mRNA expression of p27 was increased from 0 to 4 h and then decreased from 4 to 36 h (Fig. 2B). As shown in Figure 3A and B, the expression of *p27* at the protein level was significantly decreased from 12 to 36 h (*p* < 0.05).

**Table 1.** Tca8113 Cells Were Infected with *Fusobacterium nucleatum* for 4, 12, 24, and 36 h

Time (h)	G0/G1 (%)		S (%)			G2/M (%)	
	Control	F. nucleatum	Control	F. nucleatum	Control	F. nucleatum	
0	74.41 ± 0.96	74.41 ± 0.96	20.26 ± 1.05	20.26 ± 1.05	5.33 ± 0.83	5.33 ± 0.83
4	53.79 ± 0.97	52.75 ± 1.73	32.70 ± 0.64	36.23 ± 0.71^*^	11.02 ± 0.72	13.51 ± 1.03^*^
12	52.30 ± 0.37	47.33 ± 2.67^*^	27.29 ± 1.74	34.97 ± 2.47^*^	20.41 ± 1.29	17.69 ± 0.55^*^
24	67.18 ± 1.36	57.09 ± 0.13^*^	21.97 ± 0.38	26.90 ± 0.72^*^	10.86 ± 1.23	16.02 ± 0.69^*^
36	73.21 ± 1.61	61.36 ± 1.23^*^	25.54 ± 1.25	23.70 ± 1.20	1.25 ± 0.75	14.95 ± 0.48^*^

The cell cycle distribution is shown as the cell numbers in each phase (%). The data are presented as the mean ± standard deviation of three independent assays. ^*^Significant difference (*p* < 0.05) compared with the control group.

### Expression of Ku70 and p53

We checked the expression of Ku70 (Figs. 2D and 3A, D). Interestingly, the mRNA and protein expression of Ku70 were continuously downregulated from 4 to 36 h (*p* < 0.05), which suggested cell repair ability might be weakened within the infection time. We also checked the expression of p53. As shown in Figures 2 and 3, the mRNA and protein expression of wild p53 (Figs. 2C and 3A, C) was continuously downregulated from 12 to 36 h (*p* < 0.05).

### Overexpression of Ku70 weakened the cell proliferation ability by regulating the expression of Ku/p53

**FIG. 4.**
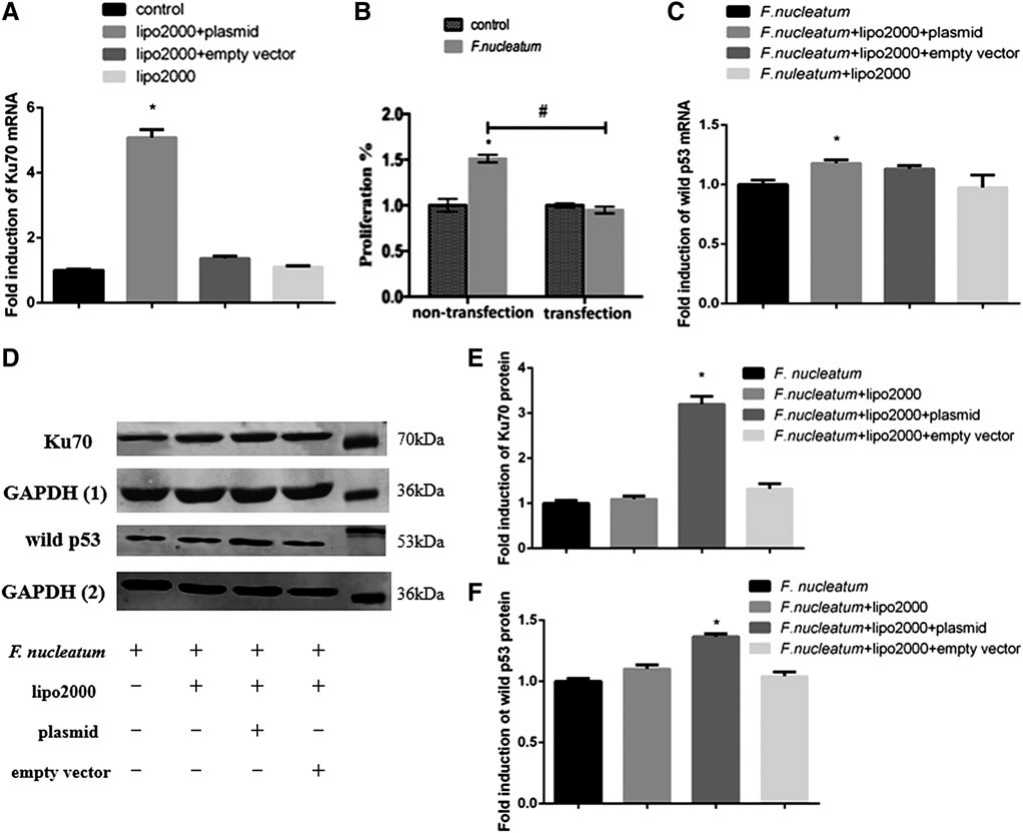
The efficiency of transfection was confirmed by qPCR **(A)**. The effect of Ku70 overexpression on cell proliferation was tested **(B)**. After overexpression with Ku70, the cells were infected with *F. nucleatum* again and the mRNA expression of wild-type *p53* is shown **(C)**. The fold inductions are presented as the mean ± standard deviation of three independent assays. *Significant difference (*p* < 0.05) compared with the control group. ^#^Significant difference (*p* < 0.05) compared between the tested groups. After transfected by Ku70, the expression of Ku70 **(D, E)** and wild-type p53 **(D, F)** in response to *F. nucleatum* was explored at the protein levels. The fold inductions are presented as the mean ± standard deviation of three independent assays.

As shown in Figure 4A, the mRNA expression of *Ku70* was significantly increased compared to the control groups (*p* < 0.05). After infection with *F. nucleatum*, the expression of Ku was still overexpressed in the transfected group (Fig. 4D, E). Accordingly, the previously increased cell proliferation ability was balanced out (Fig. 4B) with the upregulation of wild p53 (Fig. 4C, D, F).

## Discussion

The role of *F. nucleatum* in cancer has been revealed in recent years. As investigated, *F. nucleatum* is abundantly detected in colorectal cancer with the detection rate from 10% to 90% and is considered as the significant contributor to colorectal cancer (Rubinstein *et al.*, 2019). *F. nucleatum* expresses high levels of fusobacterium adhesion A (FadA) and activates oncogenic signaling to promote colorectal cancer (Rubinstein *et al.*, 2019). Evidences above implicate the possible tumorigenic role of *F. nucleatum* in other types of cancer.

As one of major periodontal pathogens in oral cavity, *F. nucleatum* appears to be reasonably associated with oral cancer (Whitmore and Lamont, 2014). Coinfection with *Porphyromonas gingivalis* and *F. nucleatum* was found to promote OSCC development by regulating the TLR2-IL-6-STAT3 axis in a chemical-induced tumorigenic mice model. Similar to our findings, tongue epithelium-derived oral cavity SCC cell lines gained significantly increased proliferation ability after coincubation with periodontal pathogens including *F. nucleatum* (Binder Gallimidi *et al.*, 2015).

We presently found that *F. nucleatum* caused cellular DNA damage with increasing upregulation of γH2AX. As known, the quantity of *F. nucleatum* can be associated with the presence of cancer. In this study, *F. nucleatum* infection at a higher MOI of 500 induced significant increased expression of γH2AX than at an MOI of 200 as well as noninfected control (shown in Supplementary Fig. S1). Similarly, higher amount of *F. nucleatum* in saliva was found in patients with colorectal cancer (Guven *et al.*, 2019). Interestingly, we have also recently reported that *F. nucleatum* was highly detected in OSCC tissues comparing to normal tissues and its presence was correlated with subgingival plaques (Chang *et al.*, 2018).

DNA damage has recently been critically reviewed and found to be closely related to the hallmarks of cancer (such as sustained proliferative signals) and is sustained during each developmental stage of cancer (Gorgoulis *et al.*, 2018). Thus, we chose a cell line of tongue squamous cell carcinoma to initially explore the effect of DNA damage caused by *F. nucleatum* in OSCC. For further study, normal cells (immortalized cells and primary cells) and other OSCC cell lines should be included to better understand the role of DNA damage in OSCC initiation and development.

The continuously increased expression of γH2AX and decreased expression of Ku indicated enhanced DNA damage with insufficient DNA repair. Meanwhile, we suggested that the increased cell growth was to some extent attributed to the dampened repair mechanism. To our knowledge, this is the first time to demonstrate that the serious DNA damage caused by *F. nucleatum* without timely repair causes an abnormal proliferation ability in OSCC cells.

In the present study, generally, the percentage of cells in the S phase was significantly increased in response to DNA damage. The downregulation of p27 was seen in this study. In accordance with our results, several studies have also revealed that downregulation of p27 causes cell cycle arrest in S phase and an enhanced cell proliferation ability (Keles *et al.*, 2003; Menchon *et al.*, 2011; Li *et al.*, 2012).

We explored the expression of Ku70 and p53, the major proteins involved in regulating NHEJ repair. Once cells are damaged, the DNA-binding domain of Ku70 is acetylated while the Ku70-dependent suppression of p53 expression is abrogated. That is, the expression of Ku70 is reduced while the expression of p53 is increased (Lamaa *et al.*, 2016). However, contrary results were shown in the present study in that the expression of Ku70 and wild p53 were synchronously decreased in association with enhanced cell proliferation ability. We hypothesize that the DNA damage caused by *F. nucleatum* at the MOI of 500 is so serious that the intracellular Ku70 is insufficient to provide prompt repair. Furthermore, without the normal regulation of Ku70, wild p53 was aberrantly expressed from upregulation to downregulation along with a possible mutation. Similar to our findings, other researchers have found a positive correlation between Ku70 and p53 changes in colon cancer (Komuro *et al.*, 2002; Lu *et al.*, 2015). At the same time, the results of the present study support the hypothesis that deletion of Ku70 or Ku80 increases the underlying damages and limits the postinjury response mechanisms mediated by p53 (Khanna and Jackson, 2001; Podhorecka *et al.*, 2010).

To further verify the pathway, we overexpressed Ku70 by transfection. As expected, the increased cell proliferation ability was inhibited with upregulation of wild p53. Thus, combined with the results above, we concluded that the possible promoting role of *F. nucleatum* in OSCC relied on the Ku70/p53 pathway after causing DNA damage. Ku70 is known as the key component of classic NHEJ that plays a critical role in detecting DSBs (Puebla-Osorio *et al.*, 2014). Thus, in the present study, we focused the specific role of Ku70 in DNA damage caused by *F. nucleatum* infection. However, another model is clearly needed to explore the role of Ku70/80 heterodimer under *F. nucleatum* infection.

In general, several deficiencies remain that need further investigation. *F. nucleatum* ATCC 25586 is the strain of *F. nucleatum* that was applied in the present study. Other subspecies of *F. nucleatum* and nonpathogens should be included in future studies, which will facilitate the understanding of the specific role of *F. nucleatum* in OSCC (Henne *et al.*, 2018). Also, to complete the mechanism of the Ku70/p53 pathway, the upstream and downstream molecules need further exploration. Moreover, in addition to cell proliferation, *F. nucleatum* infection is involved in the regulation of cell invasion and metastasis by affecting proteinase production through the activation of p38 mitogen-activated protein kinase (Uitto *et al.*, 2005). The possible effect of DNA damage caused by *F. nucleatum* on cell invasion and metastasis is needed to be further explored. Nevertheless, we demonstrated a positive role of *F. nucleatum* in causing DNA damage that promoted cell growth and enriched the mechanisms of OSCC development.

## Supplementary Material

Supplemental data
