# Supplementary material for: Fusobacterium nucleatum Caused DNA Damage and Promoted Cell Proliferation by the Ku70/p53 Pathway in Oral Cancer Cells
Source: DNA Cell Biol. 2020 Jan 8;39(1):144–51. doi: 10.1089/dna.2019.5064 (PMC6978777; doi:10.1089/dna.2019.5064)
Supplement: Supplemental data [file Supp_FigS1-TableS1.pdf]

Supplementary Data

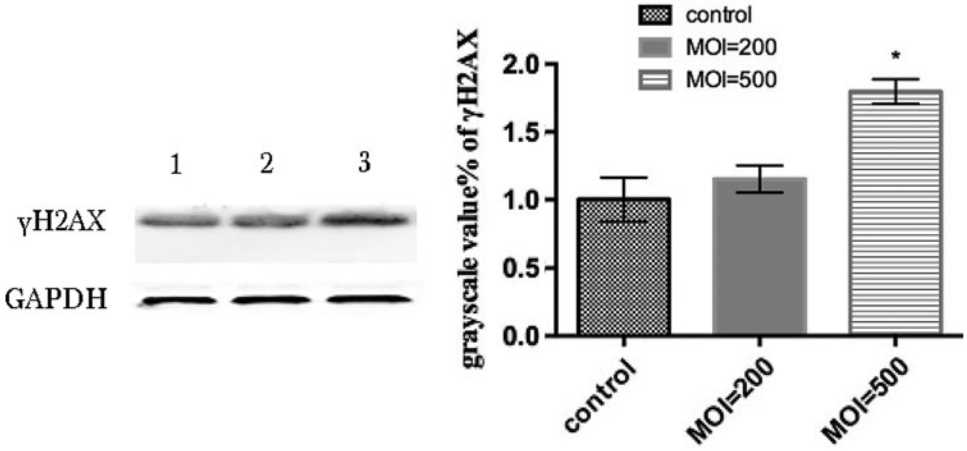

SUPPLEMENTARY FIG. S1. Tca8113 tongue squamous cell carcinoma cells were infected with *Fusobacterium nucleatum* at the MOI of 200 and 500. As shown, at the MOI of 500, the protein expression of γH2AX was increasingly upregulated compared to the control and cells infected with *F. nucleatum* at the MOI of 200. The data are presented as the mean ± standard deviation of three independent assays. *Significant difference ($p < 0.05$) compared with other groups.

Supplementary Table S1. Primers for Quantitative Real-time Polymerase Chain Reaction

Gene	*Primer sequence*	*Size (bp)*
Ku70	F: CCACAGGAAGAAGAGTTGGA	132
	R: CTGCTCTGGAGTTGCCATGA	
wild *p53*	F: GGCCCACTTCACCGTACTAA	156
	R: GTGGTTTCAAGGCCAGATGT	
p27	F: CCCTGAACGGAGCTGAAGTC	133
	R: TAACCGCGCAGCAGATAGTC	
GAPDH	F: GCACCGTCAAGGCTGAGAAC	138
	R: TGGTGAAGACGCCAGTGGA	
